# Peri-operative interventions producing better functional outcomes and enhanced recovery following total hip and knee arthroplasty: an evidence-based review

**DOI:** 10.1186/1741-7015-11-37

**Published:** 2013-02-13

**Authors:** Mazin S Ibrahim, Muhammad A Khan, Ikram Nizam, Fares S Haddad

**Affiliations:** 1 Department of Trauma and Orthopaedics, University College Hospital, 235 Euston Road, London, NW1 2BU, UK

**Keywords:** Knee, hip, arthroplasty, functional outcomes, recovery of function, surgical interventions, rehabilitation.

## Abstract

The increasing numbers of patients undergoing total hip arthroplasty (THA) or total knee arthroplasty (TKA), combined with the rapidly growing repertoire of surgical techniques and interventions available have put considerable pressure on surgeons and other healthcare professionals to produce excellent results with early functional recovery and short hospital stays. The current economic climate and the restricted healthcare budgets further necessitate brief hospitalization while minimizing costs.

Clinical pathways and protocols introduced to achieve these goals include a variety of peri-operative interventions to fulfill patient expectations and achieve the desired outcomes.

In this review, we present an evidence-based summary of common interventions available to achieve enhanced recovery, reduce hospital stay, and improve functional outcomes following THA and TKA. It covers pre-operative patient education and nutrition, pre-emptive analgesia, neuromuscular electrical stimulation, pulsed electromagnetic fields, peri-operative rehabilitation, modern wound dressings, standard surgical techniques, minimally invasive surgery, and fast-track arthroplasty units.

## Background

Joint arthroplasty is a surgical procedure performed to reduce pain, improve function, and correct deformity [1]. Hip and knee arthroplasties have been found to be very effective in improving health-related quality of life [2].

Joint arthroplasty is becoming increasingly common as the population ages and clinical researchers advance medical knowledge. The full number of total hip arthroplasty (THA) and total knee arthroplasty (TKA) procedures performed annually in the UK has increased steadily over the past decade [3]. The annual report of the National Joint Registry showed that 71,672 primary hip replacement procedures and 79,516 primary knee replacements were undertaken in 2011 [4]. A similar rise in the incidence of these procedures was noted worldwide [2]. Use of total knee arthroplasty in the USA doubled from 1999 to 2008, and this increase was noticed in all age groups [5]. This rise cannot be fully explained by population growth and the obesity epidemic alone, and is probably related to the increasing incidence of sports-related knee injuries and the expanded indications for TKR [5].

Early functional recovery and discharge from hospital are important to surgeons, patients, and health administrators. The current economic climate and restricted healthcare budgets represent additional hurdles.

In 2004, Berend *et al*. found that adopting a holistic peri-operative rapid-recovery program reduced inpatient stays and readmissions following THA and TKA. However, the authors reviewed only non-operative measures, and concluded that these can be effective in speeding recovery. They suggested combining these measures with minimally invasive surgery to achieve the best possible outcomes and faster recovery [6].

The aim of this review is to provide an evidence-based summary of measures, interventions, and procedures (both surgical and non-surgical) that help to achieve early functional recovery, reduce hospital stay, and improve functional outcome following THA and TKA.

## Review

Several interventions can be used together in a multimodal fashion, or integrated into a clinical pathway to achieve better functional outcomes, enhance recovery, and reduce hospital stay. These can be broadly classified into surgical and non-surgical interventions.

## Non-surgical interventions

### Pre-operative patient education

Pre-operative patient education has been identified as an integral component of clinical pathways for lower limb arthroplasty, and comprises group education classes covering a host of topics. These encompass: the surgical procedure and its benefits, symptoms management, operative risks and complications, the concept of early discharge planning, discharge destinations, and post-acute services. Patient education materials ranging from a simple booklet or pathway manual to educational videos have been used successfully. These clinical pathways could be an effective tool for improving patient's journey and financial outcomes following arthroplasty [7].

The patient's expectations should be considered during this process. Measures include psychological and organizational preparation, and arranging support and assistance post-operatively, which may be best achieved through an extended pre-operative consultation with anesthetist and surgeon. It is necessary to understand patient expectations in order to ensure optimal patient-reported outcome measures [8]. Conversely, dissatisfaction can result from unmet expectations [9]. There is often a discrepancy between the expectations of patients and surgeons. In a comparative study, patients had higher expectations than surgeons with regard to their ability to engage in sporting activities and exercise after undergoing arthroplasty, and patients with higher levels of disability were found to expect better outcomes than those anticipated by their surgeons [9]. Effective pre-operative education and communication are required to clarify these concepts.

There is a strong correlation between patient satisfaction and fulfillment of expectations about pain relief and functional improvement [8]. Scott *et al*. found that THA was more likely to meet important patient expectations, whereas TKA did not meet patient expectations of kneeling, squatting, and stair negotiation, despite procedural booklets explaining these limitations being given preoperatively to patients [8].

Yoon *et al*. examined how an individualized pre-operative teaching program, either by phone or in person, can affect the hospital length of stay (LOS) after TKA and THA [10]. They found that pre-operative education alone reduced LOS by 24 hours [10]. By contrast, a Cochrane Review failed to identify any significant difference in LOS between patients who had and those who had not received the education [11].

In a randomized trial, anxiety and pain levels were assessed in patients receiving pre-operative multidisciplinary standardized information sessions versus the normally provided verbal information. There was a significant pre-operative reduction in both anxiety and pain in the intervention group, but no difference in post-operative levels [12]. These findings were confirmed by McDonald *et al*., who found in a meta-analysis of nine studies that patient education moderately reduced pre-operative anxiety [11]. Daltroy *et al*. reported that a pre-operative psychoeducational program focusing on avoidance of unpleasant events reduced LOS, post-operative medication usage, and anxiety [13].

Siggeirsdottir *et al*. compared an intervention group (who received pre-operative and post-operative education by physiotherapists/occupational therapists and home visits from an outpatient team) with a control group (receiving 'conventional' rehabilitation often augmented by a stay at a rehabilitation center). They found that the intervention group had a significantly shorter hospital stay and achieved superior Oxford hip scores compared with the control group [14].

Patient education can therefore be effective in reducing LOS; however, it will be necessary to conduct further studies to evaluate the cost-effectiveness of these programs in order to justify their use.

### Pre-operative nutritional status

The effects of nutritional status on outcomes after TKA and THA are not clear. Malnutrition can lead to wound infection, delayed healing, prolonged hospitalization, and increased rehabilitation time and mortality [6,15-18].

Pre-operative biochemical indicators of nutrition status such as low albumin, total lymphocyte count and transferrin levels have been found to predict longer recovery times and hospital stay after joint arthroplasty [6,19]. Anthropometric parameters such as triceps skin fold can also be used to assess malnutrition, and has been shown to have an inversely proportional relationship with post-operative infection risk after TKA [20].

There is limited evidence showing an association between body mass index (BMI) and outcomes after arthroplasty. Although a low BMI in older patients was found to have a weak correlation (*r *= 0.246) with increased LOS after TKA in one study [21], Husted *et al*. did not find BMI to be a predictor of LOS in 712 patients undergoing TKA and THA [22]. Morbidly obese patients may present technical difficulties, such as requirement for special equipment, difficulties in pre-operative positioning and in anesthesia, and presence of excessive subcutaneous fat making the initial approach to a joint such as the hip more difficult. The cumulative effect of these factors may increase operative time and intra-operative blood loss in patients with a BMI of 40 m^2^/kg and over [23,24]. However a high BMI has not been shown to delay either early functional recovery from surgery or discharge from hospital following THA [23,24].

Pre-operative anemia can affect hospital LOS. The prevalence of anemia in patients undergoing TKA and THA is estimated at 25% [25] There is ample evidence that a low pre-operative hemoglobin (Hb) level increases transfusion requirement, infection risk, and LOS after joint arthroplasty [25-27] The absolute blood loss itself during surgery does not seem to delay discharge [21], reinforcing the notion that it may well be the patients starting point (the pre-operative Hb level) which is a more important determinant of LOS. Interestingly, a recent study did not find any correlation between postoperative Hb and early physical function recovery after TKA and THA [28], suggesting that factors other than fatigue may be responsible for delaying discharge in patients with anemia.

There may be a role for pre-operative erythropoietin and iron supplementation in patients with anemia who are about to undergo major orthopedic surgery. Iron therapy has been shown to reduce blood-transfusion rates and incidence of postoperative infection significantly, although its effects on LOS have been less clear-cut [29,30].

Pre-operative nutritional assessment may be necessary in a selected group of patients who are morbidly obese and severely malnourished. Correction of pre-operative anemia is an important measure that is gaining popularity in the orthopedic community and can potentially improve outcomes for both TKA and THA.

### Pre-emptive analgesia

Pain is a distressing symptom that can affect functional recovery, reduce patient satisfaction, and prolong hospitalization. Patient anxiety related to pain perception is another factor that can affect outcome.

The role of pre-emptive analgesia in reducing postoperative pain is still controversial. Mallory *et al*. compared three multimodal analgesic regimens [31], all using peri-operative intravenous dexamethasone and ondansetron. The first included: discontinuation of the epidural during recovery, initiation of regular oral doses of opioids for 48 hours, and use of intravenous hydromorphone for breakthrough pain. The second and third regimens used a similar protocol that differed from that of the first, and included: discontinuation of the epidural (second) or spinal (third) during recovery; initiation of patient-controlled analgesia for 24 hours during recovery, followed by oral doses of opioid medications every 4 hours through to discharge; use of intravenous hydromorphone, similar to the first regimen; and administration of cyclooxygenase-2 inhibitors for 2 weeks preoperatively and for 10 days post-operatively. The second and third regimens resulted in significantly shorter hospital stays. Pain scores during the first and second postoperative days were significantly better for patients who received the second regimen than for those who received either of the other two options [31].

However, a systematic review of the existing evidence comparing pre-emptive to post-incisional analgesia was published in the same year, which failed to show any significant improvement in postoperative pain scores recorded within 24 h after surgery in patients who had the pre-emptive treatment. This review included pre-emptive analgesia for both orthopedic and non-orthopedic operations [32].

This method also needs to be tested in the context of fast-track pathways with hip and knee arthroplasty.

### Local infiltration analgesia

Local infiltration analgesia (LIA) is an 'enabling' process that was initially developed by Kerr and Kohan in 2008, and comprises injection of a local anesthetic mixture (ropivacain, ketorolac, and epinephrine) systematically throughout the operative field [33]. The authors evaluated 325 patients who had undergone THA or TKA, and found patients had satisfactory postoperative pain control allowing mobilization within 4 hours of surgery, early discharge, and no serious complications or side effects [33]. Although this was a non-randomized study, the methods and drug mixture used became popular for modeling future randomized clinical trials (RCTs). A later RCT involving bilateral TKAs reported effective analgesia post-operatively with LIA compared with placebo [34], but in a second study, postoperative subcutaneous bolus LIA produced no improved analgesic effect [35]. Another study reported reduced pain and morphine consumption, shorter LOS, and increased patient satisfaction with LIA compared with no LIA after TKA [36]. However, in a different study, LIA was not found to provide additional analgesic or outcome benefits in the context of a comprehensive multimodal analgesic approach [37].

LIA can therefore be useful in efforts to enhance functional recovery and reduce the LOS, however further research is required in this field.

### Neuromuscular electrical stimulation

Neuromuscular electrical stimulation (NMES) involves applying transcutaneous current to neuromuscular junctions in order to stimulate muscle contractions [38,39]. It is used in both prehabilitation and rehabilitation programs for TKA, and as an adjunct to these programs to strengthen quadriceps muscle function [38,39].

Muscle stimulation failure and pre-operative muscle atrophy are factors contributing to quadriceps muscle weakness, especially in patients with osteoarthritis. Further weakness following surgery can affect the patient's overall functional recovery [38]; evidence shows that quadriceps muscle strength is reduced by 85% after TKA [40] and by 30 to 40% after THA [41].

A systematic review that examined the effectiveness of NMES in the context of muscle strengthening in TKA failed to draw any conclusion, because the included studies were biased and sample sizes were not large enough to allow for statistically robust conclusions [39]. A randomized study [38] comparing the effects of NMES on patients undergoing TKA evaluated pre-operative and post-operative quadriceps muscle strength, cross-sectional area, and clinical function against a standard rehabilitation protocol. The study found significant pre-operative gains in walking, stair-climbing, and chair-rise times in the NMES group, and similar objective functional recovery from 6 to 12 weeks post-operatively. There was no difference in the LOS between the groups [38]. Patient-reported compliance with this treatment after TKA was 99.4%, and stimulator-recorded compliance was 99% [39].

An RCT comparing the combined use of NMES and conventional physiotherapy (including extension-resisted exercises) against conventional physiotherapy alone in patients receiving total hip replacement (THR) showed significant improvement in quadriceps strength in the NMES group. There were no significant differences in walking speed or LOS between the two groups in the short term [42]. Interestingly, the extension-resisted exercises alone were found to reduce the LOS and to improve the muscle strength and functional performance significantly compared with NMES alone or conventional physiotherapy alone in a three-armed RCT [43].

This procedure should therefore be tested further in the context of fast-track recovery following TKA and THA.

### Pulsed electromagnetic fields

Local joint swelling, inflammation, and pain following THA and TKA are factors affecting patient recovery and joint function [44,45]. Holm *et al*. showed that decreased knee-extension strength, which decreases functional performance in the short term after TKA, is caused in part by post-operative knee swelling, and recommended interventions to help reduce joint swelling and improve functional recovery [46].

Use of pulsed electromagnetic fields (PEMFs) is a safe and non-invasive treatment to facilitate endogenous bone repair and reduce inflammation [45,47]. PEMFs act as an adenosine agonist on the A2a receptors of inflammatory cells. This has the strongest anti-inflammatory effect, and can reduce joint swelling and inflammation, the need for analgesics, and the time to functional recovery. This approach to treatment is also well accepted by patients [44,47].

Many studies have shown a positive effect of PEMF after knee surgery [44,47,48], but there are limited data on the effect of PEMFs after hip arthroplasty. A randomized double-blinded study on patients who had undergone revision hip surgery found that PEMFs helped to improve functional recovery and restore bone stock [45].

Another study showed that PEMFs reduced the use of non-steroidal anti-inflammatory drugs, improved functional recovery, and were well accepted by patients. No side effects were reported by patients who had undergone knee arthroscopy [47]. Similar findings were reported for patients who had undergone arthroscopic anterior cruciate ligament repair [48].

This treatment may be considered for enhanced rapid recovery; however, well-designed studies on use of PEMFs and arthroplasty are required.

## Peri-operative rehabilitation programs

Although a recent meta-analysis failed to establish the effectiveness of post-discharge physiotherapy exercises following THA in improving function, quality of life, mobility, range of motion, and muscle strength [49], a similar analysis supported the use of functional exercises to obtain short-term benefits after TKA [50]. Both these studies were concerned with post-discharge physiotherapy alone rather than with peri-operative physiotherapy in the context of enhanced recovery. Bandholm and Kehlet proposed changing the focus to earlier and more intensive physiotherapy exercise after THA and TKA (fast-track physiotherapy exercise), depending on evidence-based results [51].

Peri-operative rehabilitation is pivotal for accelerated recovery and reduced LOS after joint arthroplasty. There are several different pathways and protocols in use.

A pathway-controlled fast-track program was compared with standard rehabilitation after TKA, and was found to enhance recovery based on the American Knee Society Score and the Western Ontario and McMaster Universities Osteoarthritis (WOMAC) Index, to reduce intake of concomitant analgesic drugs, and result in a shorter LOS and reduction in adverse events [52]. This program included the patient getting up on day 0 and climbing stairs on day 2, and used positive verbal encouragement and competitive comparative patient progress [52]. A similar program providing physiotherapy on day 0 as part of a multimodal approach was compared with conventional care, and was found to provide similar results in reducing LOS and opioid consumption while maintaining a high level of patient safety [53].

The 'enhanced recovery program' (ERP) concept was introduced in 2008 in Glasgow, UK [54]. This multimodal rehabilitation program includes pre-operative patient education, followed post-operatively by early nutritional supplementation, early mobilization, and restricted opiate use to reduce nausea and ileus [55].

Malviya *et al*. introduced behavioral, pharmacological, and procedural modifications to the original program, and compared this with traditional rehabilitation at their institution. Their ERP involved pre-operative education, pre-operative and post-operative gabapentin administration, used of tranexamic acid and dexamethasone, LIA, and day 0 mobilization [54]. The traditional rehabilitation program involved mobilization on day 1 rather than day 0. This study showed that the ERP was associated with a significant reduction in death rate, LOS, and blood-transfusion requirements [54]. In another study, similar program was shown to reduce hospital stay even in patients with an American Society of Anesthesiologists (ASA) grading of 3, a pre-operative hemoglobin concentration of less than 14, and a BMI of over 30 [55].

The Norwich Enhanced Recovery Programme (NERP) is another program that was shown to be successful in reducing hospital stay (LOS = 3.5 days) with minimal complications after TKA and THA [56]. Patients using NERP are instructed by physiotherapists or nurses to mobilize 4 to 6 hours post-operatively under LIA cover, and this was shown to significantly reduce hospital stay and produce better pain scores [56].

Yoga may also be added to these programs. A study comparing conventional physiotherapy alone with conventional physiotherapy and yoga after TKA showed significant improvements in pain, stiffness, and functional WOMAC scores in the yoga group [57].

Extensive and multimodal peri-operative rehabilitation programs are effective in enhancing patient recovery after hip or knee arthroplasty.

### Modern wound dressings

Wound dressing is an integral part of the patient's journey following joint arthroplasty. It protects the newly formed tissue, allows for postoperative wound assessment, and absorbs exudates [58]. However, there are concerns about the use of traditional adhesive dressings, (for example, Mepore), with regard to wear time, number of changes, blistering, and the effect on LOS [59].

The National Institute for Health and Clinical Excellence (NICE) guidelines suggest that no clinical evidence exists to support one dressing over another [58]. A prospective audit comparing two types of wound dressings as part of an ERP in a district hospital found that traditional adhesive dressings (Mepore) resulted in a significantly shorter wear time and more dressing changes compared with the modern dressing (Aquacel Surgical), and the latter was associated with significantly less blistering. There was no difference in LOS between the groups [59].

This modern dressing design uses a viscoelastic hydrocolloid outer layer to accommodate joint motion and a highly absorbent inner hydrofiber layer designed to cope with potentially large amounts of wound exudates [59].

Dressing-related complications can affect cost and recovery time in patients following THA and TKA. More randomized controlled studies on ERPs will be necessary to assess the various types of dressings. NICE recommends a comparative study to compare the island-interactive dressings to standard adhesive dressings [58].

An RCT compared dressings covering the foot and calf in combination with LIA to assess degree of recovery after TKA. The compression bandage was found to significantly reduce pain in the first 8 hours after surgery compared with the group treated with a non-compression bandage. No difference in LOS was seen [60]. Interestingly, a better range of movement on discharge and lower LOS in the hospital was associated with the use of a similar compression bandage compared with crepe bandage was reported by Charalambides *et al*. [61].

## Surgical interventions

### Conventional surgery

#### Knee joint

There are several approaches for surgery on the knee joint.

• Standard medial parapatellar approach. This is the most popular approach to the knee in TKA. It provides excellent exposure of the knee joint, but violates the extensor mechanism and blood supply to the patella, which can affect early rehabilitation [62-64].

• Midvastus approach. Engh *et al*. described the midvastus approach in 1997. It involves opening an interval into the midsubstance of the vastus medialis while preserving its attachment to the quadriceps tendon in order to preserve function [62,65]. The advantages of this approach include increased patellofemoral stability, increased postoperative quadriceps control, and decreased scarring in the quadriceps tendon. The mid-vastus approach has been shown to significantly improve flexion and visual analogue pain scores in the first postoperative week with no effect on complication rates or quadriceps function [62]. However, the inadequate exposure to the knee in this approach is considered a disadvantage [65].

• Subvastus approach. This approach was described by Hofmann *et al*. [66]. It preserves the quadriceps mechanism [63], and involves splitting the vastus medialis at the intermuscular septum. It was reported that this approach is associated with rapid rehabilitation, better pain scores, and fewer lateral releases. However, it compromises exposure, especially in obese patients and those with limited flexion [64]. One study showed that quadriceps strength was significantly better in the subvastus group in the early postoperative period, but not during long-term follow-up after TKA [63]. Another study showed no differences in knee scores in the early postoperative period, except for slightly less extension lag in the subvastus group [67]. Patellar vascularity was not compromised nor was there any difference in anterior knee pain between the subvastus and medial parapatellar approaches [68].

More research is required in this field to determine the superiority of any of these approaches in relation to fast-track protocols.

#### Hip joint

Many surgical approaches to expose the hip joint have been described. The posterior approach is used most commonly [69]. Others include the anterolateral, direct lateral, and anterior approaches. The literature lacks comparative evidence on these approaches with regard to enhanced recovery; however a recent review comparing minimally invasive and conventional surgery was inconclusive [70]

### Minimally invasive surgery

#### Knee joint

Bonutti defined minimally invasive surgery (MIS) on the knee as involving a skin incision of less than 140 mm in length, a smaller quadriceps incision, subluxation of the patella rather than eversion, and no dislocation of the tibiofemoral joint [71]. This technique has become more popular over the past few years, although the results, compared with the conventional procedure, have been controversial. However, patients are more knowledgeable today, and expect a smaller incision, a better cosmetic outcome, and early rehabilitation [72].

One study compared conventional TKR, MIS, and computer-assisted minimally invasive knee arthroplasty, and found no significant difference in the postoperative WOMAC score, knee society score or the frequency of early complications between the three operations [72].

#### Hip joint

MIS in THA involves an incision length of 100 to 120 mm or less [73] with minimal soft-tissue damage [74]. Variable evidence in the literature exists with regard to the advantages and complications of this approach compared with the standard approach. The reduction in soft-tissue trauma during dissection is the main advantage of this approach, which in turn results in reduced postoperative pain, a smaller scar, greater mobility, reduced LOS, and less frequent requirement for blood transfusion [74].

Khan *et al*. described the mini-incision posterior approach in 2006, comparing it to the conventional posterior approach. A significant reduction in average blood loss and shorter mean LOS were achieved with use of the less invasive approach [75].

There are two types of MIS for THA: single-incision and two-incision procedures [74,76]. A comparison of these two approaches showed the two-incision approach to have significantly less time to ambulation and shorter hospital stay, but a longer operation time and more complications [76], hence this approach has since fallen out of favor.

Currently, there is growing interest in using the direct anterior approach to the hip, as described by Kennon *et al*. in 2003 [77]. This approach is thought to reduce rehabilitation time because it minimizes soft-tissue trauma [23]. However, there is a significantly longer learning curve for the surgeon, and more complications may be encountered [23,77].

A recent literature review failed to show that MIS alone results in accelerated recovery or that it reduces soft-tissue trauma compared with conventional surgery [70].

The results of MIS are promising; it can speed recovery and shorten hospital stay, especially in combination of other modalities of enhanced recovery. However, it is crucial that the goal is to minimize soft-tissue trauma rather than the length of the skin incision, and that any such procedure allows sufficient access so that component positioning and fixation are not jeopardized and that there is no adverse effect on long-term outcome. This will avoid MIS from becoming maximally destructive surgery. More research is needed in this field.

## Implant alignment and rotation in total knee arthroplasty

Malalignment and incorrect rotation can result in abnormal wear, premature loosening, and patellofemoral problems [78]. LOS was reduced by 2 days in patients with low cumulative error scores (less than 6 degrees) or with low femorotibial mismatches (between -2 and +2 degrees) compared with patients with abnormal values. However, the sample size in this study was small, which may affect the strength of this conclusion. Notwithstanding, these parameters are important in any effort to produce better functional outcomes and reduce LOS [78].

## Fast-track arthroplasty unit

Fast-track arthroplasty units (FTAUs) incorporate rapid recovery pathways designed to optimize multimodal peri-operative care and accelerate early functional recovery after TKA and THA. Their use is gaining momentum around the world, because they aim to reduce hospital costs by directly reducing LOS and peri-operative morbidity. In the current economic climate, these advantages cannot simply be ignored [79]. These units could be used in a multidisciplinary pre-operative patient clinic with multimodal pain treatment and early mobilization (4 to 6 hours post-operatively), when used in association with well-defined functional discharge criteria.

Nationwide studies from Denmark have evaluated the uptake of 'fast-track' pathways for patients with THA and TKA and shown successful implementation through a multidisciplinary and multi-institutional effort, resulting in an average reduction of LOS by more than 6 days [80]. The mean LOS of patients treated with FTAUs ranged from 2.8 to 3.9 days [81]. These units are typically used in association with standard surgical approaches, including the medial parapatellar approach to the knee and the posterior approach to the hip [81].

Despite these advantages, a select cohort of patients may fail discharge in the fast-track pathway. Husted *et al*. showed that pain, dizziness, general weakness, and postoperative anemia requiring blood transfusion were the primary clinical reasons delaying discharge at 24 and 48 hours post-operatively [82]. Waiting for physiotherapy and postoperative radiographs were identified as administrative causes of delay [82]. Holm *et al*. showed that post-operative knee swelling may lead to loss of knee-extension strength and can delay early functional recovery after TKA [46]. The use of tranexamic acid and fibrin tissue adhesive has been shown to effectively reduce blood loss and transfusion requirement after TKA [83,84]. Strategies to reduce logistic and clinical problems delaying discharge could therefore potentially result in further LOS reduction. Additional research looking at improvements in these areas and their effect on outcomes is needed.

## Conclusion and future directions

Enhanced recovery, good functional outcomes, and short hospital stays following THA and TKA can be achieved through clinical pathways and protocols with multimodal interventions. A combination of the peri-operative measures discussed earlier in this review could help to achieve these goals.

The adoption of multimodal pathways by dedicated elective centers could lead to better functional outcomes and reduce the overall cost associated with hip and knee arthroplasty. Multicenter RCTs should be used to compare outcomes from these dedicated centers to services provided at conventional hospitals. TKA and THA might even be performed as outpatient surgeries at these centers if patients could depend on the peri-operative interventions described above.

## Abbreviations

BMI: Body mass index; EPR: Enhanced recovery program; FTAU: Fast-track arthroplasty units; Hb: Hemoglobin; LA: local anesthetic; LIA: Local infiltration analgesia; LOS: length of stay; MIS: Minimal invasive surgery; NICE: National Institute for Health and Clinical Excellence; NMES: Neuromuscular electrical stimulation; NERP: Norwich Enhanced Recovery Programme; THA: Total hip arthroplasty; THR: Total hip replacement; TKA: Total knee arthroplasty; TKR: Total knee replacement; WOMAC: the Western Ontario and McMaster Universities Osteoarthritis Index.

## Competing interests

The authors declare that they have no competing interests.

## Authors' contributions

MSI conducted the main literature search, wrote the manuscript, and undertook the final editing and revision of the manuscript. MAK helped in the literature search, and the writing, editing, and revising of the manuscript. IN helped in writing the manuscript and took an editorial role. FSH is the senior author and expert in the field, who helped in writing the manuscript and was the main editor for the manuscript, directed the literature search, and advised on the contents of the manuscript. All authors have read and approved the manuscript for publication.

## Authors' information

FSH is a Consultant Orthopaedic And Trauma Surgeon at University College London Hospitals. He is Professor of Orthopaedics and Sports Surgery at the Institute of Sport, Exercise and Health, University College London, UK. IN is a Consultant Orthopaedic Surgeon in Melbourne, Australia with an interest in LIA, early mobilization, and discharge, and is currently a Visiting Arthroplasty Fellow at University College London Hospital. MI and MAK are both Clinical Research Fellows at University College London Hospital.

## Pre-publication history

The pre-publication history for this paper can be accessed here:

http://www.biomedcentral.com/1741-7015/11/37/prepub
